# Range Expansion of Moose in Arctic Alaska Linked to Warming and Increased Shrub Habitat

**DOI:** 10.1371/journal.pone.0152636

**Published:** 2016-04-13

**Authors:** Ken D. Tape, David D. Gustine, Roger W. Ruess, Layne G. Adams, Jason A. Clark

**Affiliations:** 1 Institute of Northern Engineering, University of Alaska, Fairbanks, United States of America; 2 U. S. Geological Survey, Alaska Science Center, Anchorage, United States of America; 3 Institute of Arctic Biology, University of Alaska, Fairbanks, United States of America; University of Sydney, AUSTRALIA

## Abstract

Twentieth century warming has increased vegetation productivity and shrub cover across northern tundra and treeline regions, but effects on terrestrial wildlife have not been demonstrated on a comparable scale. During this period, Alaskan moose (*Alces alces gigas*) extended their range from the boreal forest into tundra riparian shrub habitat; similar extensions have been observed in Canada (*A*. *a*. *andersoni*) and Eurasia (*A*. *a*. *alces*). Northern moose distribution is thought to be limited by forage availability above the snow in late winter, so the observed increase in shrub habitat could be causing the northward moose establishment, but a previous hypothesis suggested that hunting cessation triggered moose establishment. Here, we use recent changes in shrub cover and empirical relationships between shrub height and growing season temperature to estimate available moose habitat in Arctic Alaska c. 1860. We estimate that riparian shrubs were approximately 1.1 m tall c. 1860, greatly reducing the available forage above the snowpack, compared to 2 m tall in 2009. We believe that increases in riparian shrub habitat after 1860 allowed moose to colonize tundra regions of Alaska hundreds of kilometers north and west of previous distribution limits. The northern shift in the distribution of moose, like that of snowshoe hares, has been in response to the spread of their shrub habitat in the Arctic, but at the same time, herbivores have likely had pronounced impacts on the structure and function of these shrub communities. These northward range shifts are a bellwether for other boreal species and their associated predators.

## Introduction

Temperatures in the Arctic increased rapidly during the 20^th^ century following centuries of cooling [1,2], and the resulting landscape changes, including increased vegetation productivity and the expansion of shrubs, have been widespread [3–5]. The effects of warming and landscape changes on tundra wildlife, in comparison, are poorly documented and limited in spatial or temporal extent. Arctic vegetation in Alaska has been altered by 20^th^ century warming, yet little affected by direct human impacts, so the region provides a setting to examine the effects of altered habitat on wildlife. Here, we review historical sources across northern Alaska and the pan-Arctic to examine over a century of climatic influence on shrub habitat and moose (*Alces alces*). We focus our study on moose in the Alaskan tundra because their shrub habitat is known to have increased, and because their large size, unmistakable appearance, and importance as a source of protein for people lent them to historical documentation.

In northern and western tundra regions of Alaska, the lack of moose during the 19^th^ and early 20^th^ century was tentatively attributed to hunting by indigenous peoples and miners, and the subsequent expansion of moose has been associated with human emigration from inland regions and the resulting reduction in hunting [6,7]. However, increasing temperatures since the mid-19^th^ century have led to widespread expansion of moose’s shrub habitat in the Arctic [8–11], which could potentially supersede hunting reductions as the cause of moose establishment in the Alaskan tundra. To evaluate whether current moose presence in the Alaskan tundra is due to 20^th^ century warming and expanded shrub habitat, we used the change in cumulative summer warmth (thaw degree days) from 1850 to 2009, combined with empirical correlations between thaw degree days and shrub height, to estimate riparian shrub height starting in 1860. We compare these reconstructions of shrub height to the habitat preferences for moose [12] to evaluate whether suitable habitat existed during periods when moose were absent from the Alaskan tundra. We discuss shrub habitat expansion alongside other potential factors, such as hunting and wolf predation, in facilitating moose expansion into tundra regions.

### Moose Distribution and Habitat

Moose are the largest member of Cervidae and occupy a diversity of north temperate ecosystems. Although predation, disease, and weather influence population dynamics, suitable climate and habitat facilitate the establishment and persistence of populations, thereby shaping the regional distribution of moose [13]. The northern edge of moose distribution generally follows latitudinal treeline, occasionally extending northward into tundra along major riparian corridors [14,15].

Moose are mainly associated with early-successional habitats and selectively feed on relatively high-quality riparian shrubs, particularly willow (*Salix* spp.). In tundra and ecotonal regions, availability of forage shrubs above the snow is limiting [16,17]. Moose also utilize dense woody vegetation as cover to reduce detection by predators [18,19], and as protection during wolf attacks [20,21]. Moose on Alaska’s North Slope (*Alces alces gigas*) spend 80–90% of their tracked distance during winter in habitats with shrubs taller than 1 m, and the remainder on frozen riverbeds between thickets [12]. Longer winters and correspondingly shorter shrubs coastward [22] reduce habitat suitability and likely explain why moose distribution ends some distance south of the coast in tundra regions of Alaska, Canada, and Siberia.

**Fig 1:**
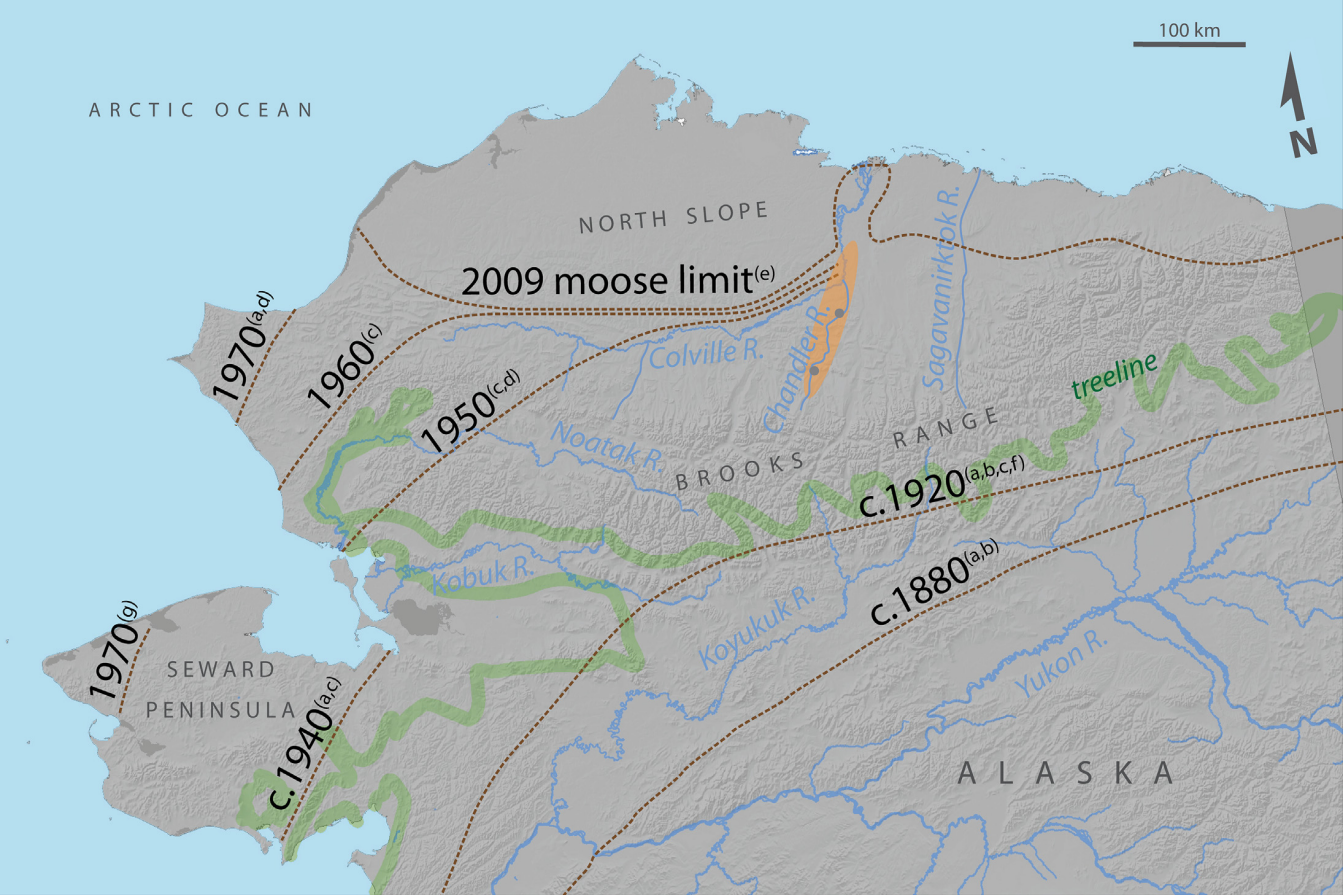
Changes in moose distribution (dashed lines) in northern Alaska since 1880 (exceptions listed in [6]). Map is inset from Fig 3. Shrub plots were distributed along the Chandler and Colville Rivers (orange ellipse), and temperature records were derived at two locations therein (gray dots) [33]. ^a^[16], ^b^[24], ^c^[6], ^d^[27], ^e^[30], ^f^[26], ^g^[31].

Moose bones (n = 18) have been recovered from eroding permafrost north of the Colville River that span 3000 ya to present, including several bones that date to the Little Ice Age [23]. The assemblage of carbon dated moose bones suggests at least a periodic presence of moose in the region during the last three millennia, though the date range on some of the more recent bones indicate that they could be modern. Evidence from archeological sites, indigenous peoples, and early explorers documents an absence of moose during the latter half of the 19^th^ and early 20^th^ century in tundra areas of Alaska [6,24–26], with few exceptions [6,27]. During the second quarter of the 20^th^ century, moose began to appear in tundra regions of northern and western Alaska (Fig 1), and similar increases were observed in northern Canada (*A*. *a*. *andersoni*) [6], western Siberia [28], and western Russia (*A*. *a*. *alces*) [29]. By the 1940s, moose populations were becoming established along the riparian shrub corridors of the Colville River and its tributaries in Arctic Alaska. An aerial population survey in late-winter of 1950 revealed 109 moose along a 90-km section of the Colville River floodplain [6]. Extensive late-winter surveys in 1970 and 1977 covering most of the North Slope (Utukok to Kongakut River) recorded between 1550 and 1700 moose [6], with approximately half of those moose residing in the middle Colville drainage [30], confirming their establishment. By the 1980s moose had colonized northwest Alaska (Fig 1) [31].

## Methods

### Estimating Shrub Height

We used the relationship between summer warmth and willow height developed by Walker [22] to estimate changes in the height of riparian willows from c. 1860 to 2009. Walker [22,32] measured the 50 tallest willows (*Salix richardsonii*) at multiple streamside sites along a temperature gradient in Arctic Alaska and derived a relationship between thaw degree days (*TDD*) and shrub height:

$$\mathrm{shrub height}\;\left(cm\right)=\;0.000341{\left(TDD\right)}^{2}-0.195\left(TDD\right)+27.7$$ Eq. 1

(R^2^ = 0.97, *P* = 0.002, 0 cm < *shrub height* < 148.8 cm). Using interpolated historical temperature data from Scenarios Network for Alaska & the Arctic Planning (SNAP) [33] to calculate thaw degree days based on mean monthly temperatures greater than 0°C (average 893 TDD in 1901–1910 vs. 1048 TDD in 2000–2009), we estimated shrub heights between 1901 and 2009 for two locations in Arctic Alaska (Fig 1). We assumed that shrub height was a function of the past 10 years of summer warmth, and therefore used an average of the previous 10 years of thaw degree days to estimate shrub heights annually since 1910. Due to the lack of observed temperature data prior to 1901, we included hindcasted temperatures for 1850–1900 generated by SNAP for the five General Circulation Models (averaged: *CMI3P/AR4 5modelavg*) that performed best over Alaska [34]. A 95% confidence interval was calculated around the predicted shrub height using Eq 1 and the original data [32]. We assumed that *Salix alaxensis*, the common floodplain willow species preferred by moose, responded to increases in cumulative warmth similarly to the willow *Salix richardsonii*, given their co-occurrence on floodplains [35] and their similarly dramatic increase in canopy volume as mean July temperature approaches 12°C [36]. In 2010, we measured heights of streamside shrubs, including the 50 tallest willows (*Salix* spp.) and Siberian alders (*Alnus viridis*, ssp. *fruticosa*) occurring within each of eight 250 m by 250 m plots along the Chandler and Colville Rivers. These two riparian corridors were selected because they have the greatest density of tall shrubs and moose north of the Continental Divide of the Brooks Range [30,37], and would likely have been the first riparian corridors with sufficient habitat for moose. We compared our predictions of shrub heights in 2009 (from Eq 1) to our measured values in 2010. Mean and standard error are reported, unless otherwise mentioned.

## Results

**Fig 2:**
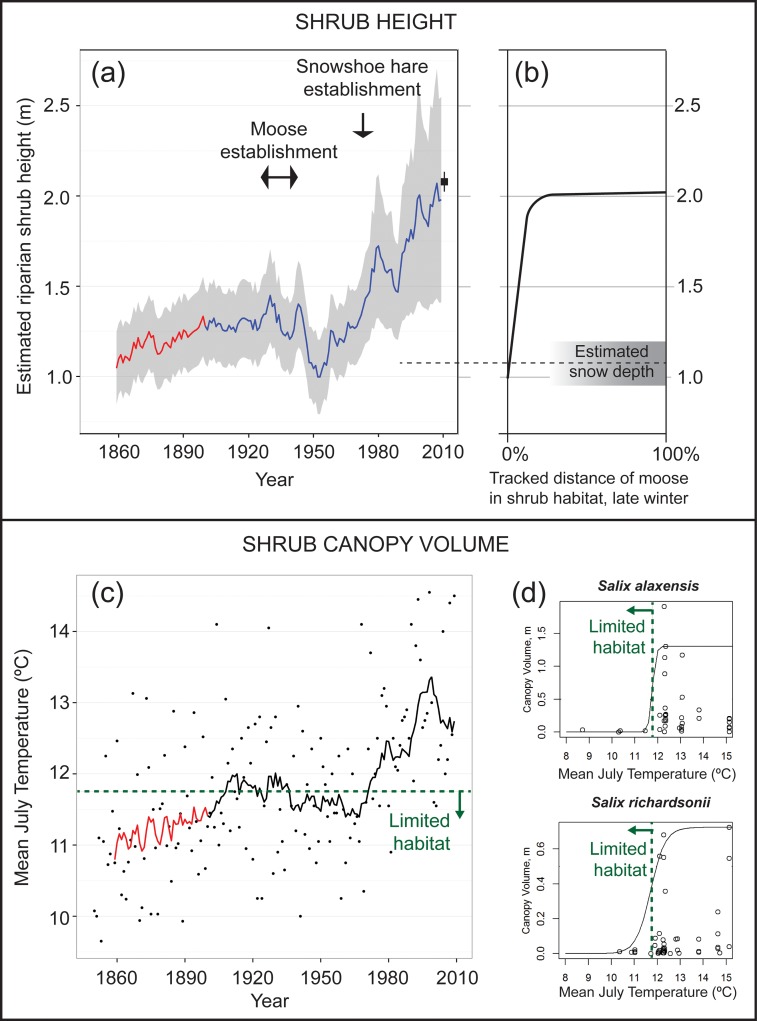
**Estimated changes in moose habitat in Arctic Alaska since 1860, including changes in riparian shrub height (a, b) and shrub canopy volume (c, d).** Moose require shrubs protruding above the snow in late winter for habitat [b; adapted from [12]], and the shrub height hindcasting (a) indicates that little to no habitat would have existed in Arctic Alaska c. 1860 (a, b). The blue shrub height line (a) uses an average of previous 10 years of thaw degree days to predict shrub height (Eq 1); shaded area indicates the 95% confidence interval; predicted height is consistent with measured shrub heights in 2010 (black box). The blue shrub height line uses interpolated observed temperatures, whereas the red line uses temperatures hindcasted from an average of the five highest-performing General Circulation Models [34]. The mean July temperatures of the late 1800s and early 1900s (solid line is average of previous 10 yrs) were less than 11.8°C (c), indicating that willow canopy volumes would have been greatly reduced (d), consistent with the absence of moose during that period (S1 & S2 Appendices). Temperature sensitivity of shrub canopy volume for *Salix richardsonii*, the species used to construct (a), is similar to that for the preferred forage species of moose, *Salix alaxensis* (d; reproduced from [36]).

Our hindcasting of shrub height, based on the strong positive relationship between streamside willow height and thaw degree days [22], indicates that the shorter and cooler growing seasons c. 1860 would have resulted in shorter willows. For streamside willows, we estimate that the 25% increase in thaw degree days along the Chandler and Colville Rivers from 1850 to 2009 is correlated with a 79% increase in shrub height (Eq 1, Fig 2A), from 1.10 ± 0.07 m in 1860 to 1.97 m ± 0.21 m in 2009. The 1.97 ± 0.21 m estimated height of tall riparian willows is similar to the measured height in 2010 of 2.08 ± 0.06 m (range = 1.86–2.37 m). Using only the interpolated observed temperatures, which start in 1901, we estimate that the 17% increase in thaw degree days along the Chandler and Colville Rivers from 1901 to 2009 is correlated with a 63% increase in shrub height, from 1.25 ± 0.05 m in 1910 to 1.97 m ± 0.21 m in 2009.

## Discussion

### Possible Causes of Moose Establishment in the Arctic

The spatial expansion and vertical growth of riparian shrubs in response to warming initiated in the 19^th^ century would have substantially increased winter moose habitat. Moose in the Alaskan tundra are limited by winter range and 80–90% of their tracked distance in winter is located in habitat with shrubs taller than 1 m [12]. The estimated tall shrub height of 1.10 m c. 1860 suggests that there was little to no moose habitat available (Fig 2A and 2B). *Salix richardsonii*, the willow species measured along a thermal gradient [22,32] and used here, responds very similarly to mean July temperature exceeding 11.8°C as does *Salix alaxensis*, both by dramatically increasing canopy volume [36]. Mean July temperature was below 11.8°C c. 1860, again indicating that little to no moose habitat existed then (Fig 2C and 2D). General agreement between the observed riparian shrub heights in 2010 (2.08 ± 0.06 m, range = 1.86–2.37 m) and those predicted by the temperature records in the region (1.97 m) lends support to the thermal gradient approach used here.

In tundra regions moose require shrubs protruding above the snow [16,17,38]. In riparian corridors, the valley topography and shrubs dampen the erosive wind events that commonly scour snow from the surrounding tundra, leading to snow depths more than twice as great in the riparian shrubs as on the surrounding tundra [39]. The mean late-winter snow depths of 0.51 and 0.57 m reported for successive years across the Kuparuk River basin of Arctic Alaska (adjacent to the Colville basin) excluded the deeper snow found in tall shrubs [40]; doubling the snow depth measured in open tundra [39], provides an estimate of average snow depth among riparian shrubs of approximately 1.1 m (Fig 2B). Little available forage would have protruded above the snow prior to moose establishment, and an increase in average shrub height from 1.1 to 2 m since 1860 would have dramatically increased late-winter forage. The increase in shrub height might have captured more drifting snow, potentially negating some of the gains in available forage, but the scant record of historical snow distribution led us to assume a variable but trendless end-of-winter snow cover amid increasing shrub heights. Finally, adult moose in Alaska stand approximately 1.9 m tall at the shoulder, or approximately 0.8 m above an average riparian shrub stand in 1860, whereas current shrubs are tall enough to obscure and obstruct moose from predators.

The only proxy record of shrub production dating to the 19^th^ century was reconstructed from sediment cores collected from the Colville River delta, and it shows a much greater increase in shrub production (as indicated by increases in fresh particulate organic matter transported to the delta) in the Colville watershed between 1850 and 1950 than after 1950 [8], when repeat photography documents an increase [41]. Floodplain riparian shrub cover in northern Alaska increased from 5% to 13% between 1950 and 2000, and logistic growth rates of shrub cover also suggest that shrub expansion was initiated c. 1875 [41]. Shrub expansion initiated c. 1850–1880 is also supported by direct and proxy air temperature records from Alaska and other Arctic locations showing that warming began between 1850 and 1880, reversing a long-term cooling or stable period before 1850, known as the Little Ice Age [1,10]. Summer warming prior to 1907 is evident from historic photos showing the retreat of glacier terminuses in Arctic Alaska [42] from their Little Ice Age maximum extents—retreat that had accelerated by 1957 [43] and continues today [44]. Increasing mean annual air temperatures 50 to 75 years leading up to the 1950s was also evident in warming permafrost borehole temperature profiles from the region [45]. Warming inferred from proxy records is consistent with temperature data generated by running GCMs backward in time, which show an increase in summer temperature between 1850 and 1950 (Fig 2A and 2C).

Our result of increasing shrub habitat linked to moose establishment can confidently be extrapolated across the North Slope and Brooks Range, where summer temperatures are comparable and there is a record of shrub habitat increase. In this region, disturbances are muted and generally confined to riparian corridors, where most shrub expansion has occurred [46,47]. In treeline and forested regions, including parts of the Seward Peninsula and Koyukuk River regions, the record of habitat change is more ambiguous, and thus the linkage between moose establishment and habitat is less reliable. We suspect that shrub expansion has actually occurred more rapidly in treeline regions than in the tundra, and this is supported by anecdotal evidence from the Kobuk River region [48]. Disturbance regimes, notably permafrost thaw and wildfire, are rare in the tundra, but are active in treeline and forested regions, and they amplify the direct effects of temperature on deciduous shrubs by initiating early successional vegetation dominated by shrubs and saplings, thus expanding moose habitat. Increasing permafrost thaw and wildfire associated with warming in the boreal forest and ecotonal regions have likely created more deciduous moose habitat [49–51] than in the tundra, but the restructuring of the boreal forest has yet to be quantified, particularly in terms of increasing moose habitat.

### Hunting Hypothesis

A previous study suggested that the lack of moose in tundra areas of Alaska during the early 20^th^ century was due to hunting by coastal and inland peoples. As people emigrated from inland tundra regions to the coast (indigenous people in the north in the 1920s, miners on the Seward Peninsula in the 1940s), hunting pressure was reduced, allowing moose to immigrate and inhabit riparian corridors of the tundra [6]. Perhaps the greatest strengths of the hunting hypothesis as it applies to northern Alaska are (1) caribou were heavily hunted by coastal communities prior to periods of low caribou abundance from 1870 to 1900, as revealed by archeological, anthropological, and early written records [25], and (2) low caribou abundance and the introduction of Western diseases [52] reduced the inland indigenous population or drove them to the coast by the 1920s, thereby reducing inland hunting pressure. There are ample accounts of indigenous peoples on foot or with dogteam in the boreal forest relentlessly following moose tracks in snow until the animal was reached and shot [53,54], and tracking may have been easier in 19^th^ century tundra environments where visibility was good and patches of tall shrub habitat were greatly reduced and limited to fragments along floodplains. Unlike for caribou, however, evidence of moose or moose hunting in tundra regions between 1800 and 1900 is scarce. Archeological sites in Alaskan tundra and treeline regions reveal a paucity of moose remains until the mid-20^th^ century, consistent with the absence of moose in historical accounts and with the minor representation of moose in northern indigenous culture and lore [6,24,27,52]. The introduction of breech-loading firearms during the mid-19^th^ century corresponded with a rapid decline in caribou populations, but also with the onset of moose expansion from its refugium in Yukon Flats [25].

**Fig 3:**
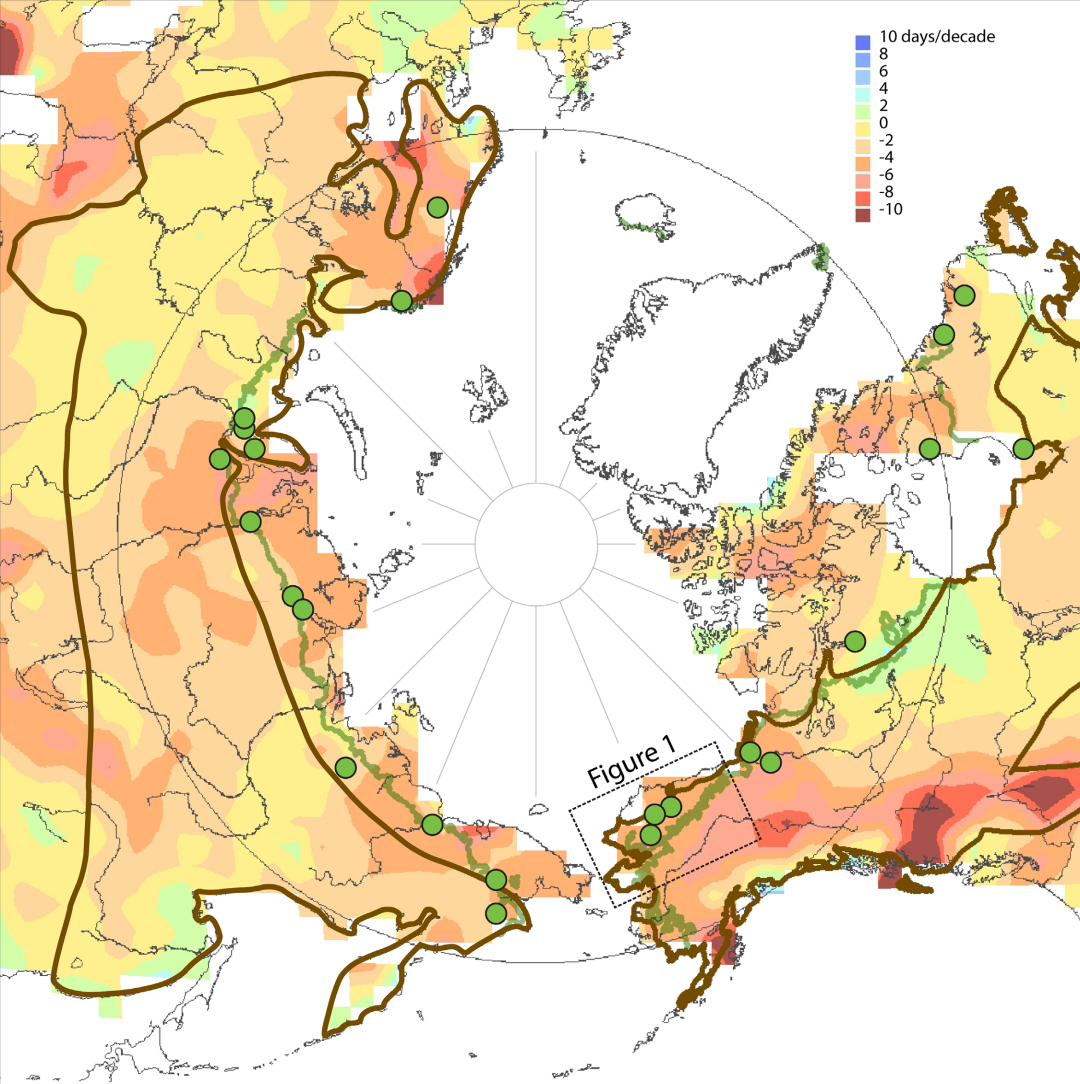
Overlapping areas of northern moose distribution (brown outline) [14,15], earlier spring onset (shaded) [74], and documented shrub expansion (green dots) [3,4]. Shaded areas indicate trends in spring snow cover duration 1972–2008 (see legend). The green line denotes treeline. Increasing shrub habitat along the northern edge of moose distribution portends northward range extension of moose across the Arctic.

The hunting hypothesis was articulated well before recognition of climate change and resulting shrub expansion in northern Alaska [9] and the pan-Arctic [3,4]. The current absence of moose along the northern Arctic coast in locations where no forest occurs (Fig 3) is due to longer winters associated with lingering sea ice and correspondingly shorter shrubs [<0.3 m; 22] that fall below the shrub-height habitat requirement [12], not hunting pressure from coastal communities. Furthermore, the snowshoe hare (*Lepus americanus*), another obligate browser with a similar distribution and habitat requirement to moose in northern North America [15], extended its range northward to the Colville River [30,55–57] during the 1970s. This shift cannot be explained by hunting reduction, but is instead likely due to ameliorating climate and increased shrub habitat [58]. The earlier arrival of moose than snowshoe hares could be due to greater shrub habitat requirements for snowshoe hares, or to faster dispersal rates of moose. We think these findings of decreased habitat during the 19^th^ century supersede hunting in explaining moose absence. Nonetheless, if the fragmentary moose habitat possibly present during the 19^th^ century had supported small numbers of moose, then the effect of hunting in the late 19^th^ century may have been to delay moose dispersal into treeline and tundra regions [25] where new shrub habitat was available. Finally, though wolf predation has been shown to limit moose abundance in many areas [59], wolf populations, like caribou, were increasing during and prior to the expansion of moose in the region [6]. Aerial wolf culling by the U.S. Fish & Wildlife Service was implemented after moose were established [60,61].

### Effects of Adding Herbivores to Expanding Shrub Ecosystems

Moose and snowshoe hare are known to have pronounced impacts on vegetation composition, structure, net primary production, and ecosystem function [62]. Exclosure experiments in other arctic locations have shown that reindeer (*Rangifer tarandus*) browsing reduced height and cover of shrubs across a variety of deciduous shrub species [63–65], while changes in shrub biomass were generally neutral or negative [66]. The 20^th^ century arrival of snowshoe hares and moose provides a natural test of the effects of adding herbivores to vegetation already responding to warming, but the preexistence of ptarmigan (mostly *Lagopus lagopus*) complicates matters. In Arctic Alaska, there is a higher probability of browsing by ptarmigan on palatable willows than browsing by moose and hare combined [66]. Lacking moose and snowshoe hare c. 1850, and with fewer and shorter shrubs, ptarmigan could have been greater in number and removing proportionally as much biomass as all three herbivores do now. In any case, the addition of moose and snowshoe hares to tundra riparian corridors in northern Alaska has not been sufficient to curb shrub expansion, but their addition may have intensified the effects of browsing on willow shrub architecture, including ‘brooming’ of stems and sprouting juvenile ‘stump’ shoots widely observed [67]. In Alaskan floodplains, heavy browsing on willows often favors establishment and dominance by alders, which fix atmospheric nitrogen and are chemically defended against herbivory [68,69]. The indirect effects of browsing increase alder abundance and nitrogen input [62,70], which substantially influences net ecosystem production, and may have contributed to the recent spread of alder in arctic ecosystems [41].

### Similarities between Late-Pleistocene and Modern Moose Expansion

The range expansion of moose into the Alaskan tundra during this recent ecological shift characterized by warmer temperatures and increased plant production resembles shifts that occurred during the Pleistocene-Holocene transition. Pleistocene steppe tundra with long cold winters and sporadic snow cover was altered by a warmer, wetter Holocene that stimulated widespread shrub expansion. Concurrent changes included the extinction of large-bodied grazers such as horse (*Equus* spp.) and mammoth (*Mammuthus* spp.) 14,000 to 11,000 radiocarbon years ago, and the arrival of humans and moose c. 13,000 radiocarbon years ago [71]. The cause of extinction of these large-bodied grazers, whether climate change or human hunting or some combination of both, has been vigorously debated, with recent evidence favoring abrupt climatic change and reduced habitat as the primary cause [72,73], which is consistent with the recent creation of shrub habitat causing establishment of moose in the tundra. The simultaneous arrival of humans and moose during the late Pleistocene warming also implies a similar minimum environmental or habitat requirement; moose required shrubs for winter forage and cover, while humans may have needed shrubs for firewood or shelter [23]. Thus, an interesting implication of our findings is that the recent colonization of Alaskan tundra areas by moose may indicate that the current shrub vegetation and climate conditions are comparable to those that were necessary for paleohuman colonization, notwithstanding other differences between the ancient and modern ecosystems.

## Conclusion

We think that climatic constraints on moose habitat c. 1850 prevented moose from colonizing tundra regions of Alaska. The combination of longer growing seasons [42] and increasing shrub habitat after 1850 [8] allowed moose to colonize tundra regions of Alaska and, later, to sustain populations hundreds of kilometers north and west of previous distribution limits. Increases in shrubs and earlier snowmelt have occurred across most of the Arctic [3,4,74], notably along the northern edge of moose distribution, so increases in abundance and extended northward distribution of moose are anticipated elsewhere (Fig 3). Indeed, the expanded shrub habitat observed across the Arctic may be contributing to observed increasing numbers of moose in parts of northern Canada [6] and northern Eurasia [28,29], as it is in Alaska.

## Supporting Information

S1 Appendix Contains data used to create Fig 2A & 2C. (XLS)

S2 Appendix Contains General Circulation Model data supporting Fig 2A & 2C. (XLSX)
