# Correction: Range Expansion of Moose in Arctic Alaska Linked to Warming and Increased Shrub Habitat

**DOI:** 10.1371/journal.pone.0160049

**Published:** 2016-07-21

**Authors:** Ken D. Tape, David D. Gustine, Roger W. Ruess, Layne G. Adams, Jason A. Clark

There are errors in the Funding Statement. The correct funding information is as follows: KDT was supported by EPSCoR NSF award #OIA-120892 and by NASA award NNX09AL03G. Fieldwork and data compilation from the Chandler/Colville Rivers was supported by NASA award NNX09AL03G Mapping Changes in Shrub Abundance and Biomass in Arctic Tundra using NASA Earth Observing System Data: A Structural Approach (PI: Mark Chopping). DDG and LGA received support from U.S. Geological Survey’s Changing Arctic Ecosystem Initiative within the Wildlife Program of the Ecosystem Mission Area. The funders had no role in study design, data collection and analysis, decision to publish, or preparation of the manuscript.

Additionally, the measured shrub height data from the Chandler/Colville Rivers that KD Tape helped to collect and was used in this study was published and archived online several months earlier, and thus should have been mentioned in the Data Availability Statement.

The complete, correct Data Availability Statement is as follows: Data used to create Eq 1 are available in Walker DA. 1985. Vegetation and environmental gradients of the Prudhoe Bay region, Alaska. Hanover, New Hampshire: Cold Regions Research and Engineering Lab. Report no. 85–14. pp. 238. Shrub height data from the Chandler/Colville Rivers are available in Duchesne, RR, MJ Chopping, and KD Tape. 2015. Capability of the CANAPI algorithm to derive shrub structural parameters from satellite imagery in the Alaskan Arctic. Polar Record. pp.1-10, available online at Oak Ridge National Laboratory Distributed Active Archive Center: http://dx.doi.org/10.3334/ORNLDAAC/1270. All other relevant data are within the paper and its Supporting Information files.
